# Utility of Absolute Quantification in Non-lesional Extratemporal Lobe Epilepsy Using FDG PET/MR Imaging

**DOI:** 10.3389/fneur.2020.00054

**Published:** 2020-01-31

**Authors:** Tatjana Traub-Weidinger, Otto Muzik, Lalith Kumar Shiyam Sundar, Susanne Aull-Watschinger, Thomas Beyer, Marcus Hacker, Andreas Hahn, Gregor Kasprian, Eva-Maria Klebermass, Rupert Lanzenberger, Markus Mitterhauser, Magdalena Pilz, Ivo Rausch, Lucas Rischka, Wolfgang Wadsak, Ekaterina Pataraia

**Affiliations:** ^1^Department of Biomedical Imaging and Image-Guided Therapy, Medical University of Vienna, Vienna, Austria; ^2^Department of Pediatrics, The Detroit Medical Center, Children's Hospital of Michigan, Wayne State University School of Medicine, Detroit, MI, United States; ^3^QIMP Team, Center for Medical Physics and Biomedical Engineering, Medical University of Vienna, Vienna, Austria; ^4^Department of Neurology, Medical University of Vienna, Vienna, Austria; ^5^Department of Psychiatry and Psychotherapy, Medical University of Vienna, Vienna, Austria; ^6^Ludwig-Boltzmann Institute Applied Diagnostics, Vienna, Austria; ^7^Center for Biomarker Research in Medicine, Graz, Austria

**Keywords:** PET/MRI, extratemporal lobe epilepsy, absolute quantification, metabolic rate of glucose, brain imaging

## Abstract

The purpose of this study was to establish a non-invasive clinical PET/MR protocol using [^18^F]-labeled deoxyglucose (FDG) that provides physicians with regional metabolic rate of glucose (MRGlc) values and to clarify the contribution of absolute quantification to clinical management of patients with non-lesional extratemporal lobe epilepsy (ETLE). The study included a group of 15 patients with non-lesional ETLE who underwent a dynamic FDG PET study using a fully-integrated PET/MRI system (Siemens Biograph). FDG tracer uptake images were converted to MRGlc (μmol/100 g/min) maps using an image derived input function that was extracted based on the combined analysis of PET and MRI data. In addition, the same protocol was applied to a group of healthy controls, yielding a normative database. Abnormality maps for ETLE patients were created with respect to the normative database, defining significant hypo- or hyper-metabolic regions that exceeded ±2 SD of normal regional mean MRGlc values. Abnormality maps derived from MRGlc images of ETLE patients contributed to the localization of hypo-metabolic areas against visual readings in 53% and increased the confidence in the original clinical readings in 33% of all cases. Moreover, quantification allowed identification of hyper-metabolic areas that are associated with frequently spiking cortex, rarely acknowledged in clinical readings. Overall, besides providing some confirmatory information to visual readings, quantitative PET imaging demonstrated only a moderate impact on clinical management of patients with complex pathology that leads to epileptic seizures, failing to provide new decisive information that would have changed classification of patients from being rejected to being considered for surgical intervention.

## Introduction

Surgical interventions in patients with intractable epilepsy are planned based on a variety of diagnostic measures, including electrophysiological testing and neuroimaging procedures (1–3). The results of epilepsy surgery are very promising, with high chance of cure, especially in temporal lobe epilepsy, resulting in significant higher seizure freedom postoperatively compared to medical treatment (4, 5). However, the localization of seizure onset zone in extratemporal lobe epilepsies (ETLE) is very difficult in non-lesional forms (6, 7) and the identification of epileptogenic zones, including definition of its boundaries, remains challenging. As such, there is a need for further development of methodologies that could provide more localized information about tissue epileptogenicity.

It is well-accepted that cortical glucose hypo-metabolism depicted by 2-[^18^F]fluoro-2-deoxy-D-glucose (FDG) PET provides valuable imaging clues with respect to the location of electrophysiologically confirmed seizure onset zones (8, 9). FDG PET imaging can be particularly useful in non-lesional patients, as it can often lateralize and even localize potentially epileptogenic cortical regions to guide intracranial electrode placement (10). However, detailed analysis of the spatial relationship between glucose hypo-metabolic areas and intracranial EEG-based seizure onset zones exhibits a complex association between functional and electrophysiological data that are difficult to entangle in the absence of absolute metabolic rate of glucose (MRGlc in μmol/100 g/min) values. Moreover, hyper-metabolic brain areas are occasionally observed during the patients' interictal states that are poorly understood and as a result are either ignored or viewed as being of questionable significance (11, 12). However, such areas might provide additional clues with respect to the location of either frequently spiking cortex (13) or might denote the presence of a network of inhibitory circuits that is activated in order to prevent the propagation of the epileptic discharge (14). This view is substantiated by the observation that ictal intracranial EEG and glucose metabolic abnormalities in the human cortex are known to match only partially (15).

Although PET imaging using FDG as the tracer of choice allows quantitative assessment of glucose metabolic rate (MRGlc), absolute metabolic rates have been rarely used in clinical routine to characterize abnormal brain areas in ETLE patients. The reason for this omission to fully embrace the potential of PET imaging in clinical applications is the requirement of arterial blood sampling, an invasive procedure not performed routinely. However, with the advent of hybrid imaging, quantification of MRGlc can be achieved non-invasively based on the simultaneous acquisition of both high-resolution anatomical as well as high-sensitive molecular functional data, yielding an image-derived arterial input function from an integrative analysis of PET and MR image data (16). Consequently, this methodological advance enables the determination of absolute regional MRGlc values in clinical routine and might provide added value during diagnostic evaluation of ETLE brain scans (16).

In the present study, we obtained regional MRGlc values from a series of patients with drug-resistant non-lesional epilepsy who were previously rejected as surgical candidates based on a comprehensive clinical work-up. The objectives of the study were as follows: (i) to establish a non-invasive clinical protocol that provides the physician with regional MRGlc values in epilepsy patients; (ii) to clarify the contribution of absolute quantification to clinical readings; and most importantly (iii) to determine whether quantitative PET imaging is able to provide sufficient new information that would allow re-classification of patients from being rejected to being considered for surgical interventions.

## Materials and Methods

### Subjects

In this prospective study we included 15 patients with drug-resistant non-lesional ETLE who were previously rejected for surgical intervention based on an extensive pre-surgical work-up at the Epilepsy Monitoring Unit of the Department of Neurology which included prolonged Video-EEG monitoring, a high-resolution 3T MRI scan using a predefined clinical epilepsy protocol (see below), formal neuropsychological testing and (in selected patients) interictal and ictal SPECT, as well as visual PET and functional MRI for lateralization of language functions.

Video-EEG monitoring was recorded for an average of 5 days. The EEG was recorded according to the international 10–20 System with bilaterally placed additional sphenoidal electrodes. The absolute spike frequency over the entire recording time and its location was assessed by visual analysis. The clinical seizure semiology was evaluated with respect to the prediction of symptomatogenic area(s) and the ictal EEG patterns were determined over the time course by location and morphology. Two board-certified epileptologists (EP and SA-W) with 20 years of experience in epileptology reviewed all clinical information and Video-EEG monitoring results.

MRI studies were assessed with respect to epileptogenic lesions including focal cortical dysplasia, hippocampal/mesial temporal sclerosis and epileptogenic tumors. Imaging was performed on a 3T MR system (Trio, Siemens, Erlangen, Germany) with an 8-channel head coil. All studies employed a high-resolution epilepsy protocol that included the following sequences: a 3D volumetric spoiled gradient-echo T1-weighted (T1-w, TR/TE 1610/3, 71 ms, resolution 1/1/1 mm) and a sagittal 3D fluid attenuation inversion recovery (FLAIR) MR sequence (TR/TE = 6,000/393 ms, TI = 2,100 ms, resolution=1 × 1 × 1 mm, matrix 512 × 512) with multi-planar reconstructions. Moreover, a T2-turbo-spin-echo MR sequence (T2-TSE, TR/TE = 3,550/113 ms, 2 mm slice thickness, matrix 304 × 320), an IR (TR/TE = 4,000/380 ms, resolution 0.9 × 0.9 × 0.9 mm) and a FLAIR sequence (TR/TE = 4,000/381 ms, slice thickness = 2 mm, matrix 448 × 512) were acquired in a coronal plane perpendicular to the hippocampus. A board-certified neuroradiologist (GK) with 10 years of experience in epilepsy imaging, who was blinded to all other clinical information, reviewed all patients' MR scans and assessed them as being non-lesional.

Based on the results of pre-surgical evaluation a suspected epileptogenic area was delineated and allocated to the corresponding brain region. If a clear assignment to a brain region/hemisphere/lobe was not possible, the seizure area was defined as non-localizable. The final decision regarding the procedure (invasive recordings or rejection from surgical procedure) was made individually for each patient based on case presentation and discussion at the institutional multidisciplinary epilepsy conference.

Patients rejected as surgical candidates underwent subsequently a brain examination on a fully-integrated PET/MRI system in the Department of Nuclear Medicine, Medical University of Vienna, Austria, between September 9, 2017 and July 1, 2018.

### PET/MRI Protocol

The study was approved by the Ethics Committee of the Medical University of Vienna and was performed in accordance with the Declaration of Helsinki (1964) including current revisions, the Austrian Drug Law and the GCP guidelines of the European Commission. Written informed consent was obtained from all subjects prior to the examinations.

Patients underwent a brain examination in the interictal state on a fully-integrated PET/MRI system (Siemens Biograph mMR, Erlangen, Germany) as described previously (16). In short, a venous line was established for the injection of the FDG tracer and support foam cushions were placed to brace the subject's head in order to reduce involuntary head movement. Initially, a 3D time-of-flight MR angiography (3D-TOF MRA) sequence was used to image the internal carotid arteries (0.5 × 0.5 × 1 mm^3^ voxel size, TE = 3.6 ms, TR = 21 ms, 6 min duration). Subsequently, FDG (5.18 MBq/kg) was injected through the venous line as a slow bolus (40 s) and a 60 min list mode PET data acquisition was initiated. During PET list mode data acquisition, a second set of T1-w and T2-w sequences were acquired in order to assess potential disease progression relative to the initial MR scan as well as for the calculation of a pseudo-CT attenuation correction map (17). List mode data was re-binned into a dynamic frame sequence (24 × 5 s, 1 × 60 s, 1 × 120 s, 1 × 300s, 1 × 600 s, 2 × 1,200 s) and each PET frame was reconstructed using the ordinary Poisson OSEM 3D algorithm (3 iterations, 28 subsets, 2 mm Gaussian filter). The history of last seizure, medication regimen and vital parameters were collected immediately before testing. In the case that a seizure occurred within 24 h before testing, the PET-scan was rescheduled. Patients were closely monitored for signs of seizure activity prior and during the PET imaging procedure and (after removal of the patient from the scanner gantry) patients were subjected to a post-imaging examination to determine whether a subclinical seizure episode could have happened. None of the patients showed seizure semiology during the PET scans nor did the post-imaging examination indicate signs of a subclinical epilepsy episode.

### Non-invasive Quantification of Glucose Metabolic Rate

We have previously developed a methodology that enables the acquisition of a non-invasive image-derive input function (IDIF) based on simultaneously acquired structural and functional data obtained using a fully integrated PET/MR system (16). This methodology takes advantage of the spatial correspondence between structural information of the internal carotid artery (ICA, derived from the MRA sequence) and the PET tracer concentration distribution in and around the ICA (derived from the FDG PET image) in order to apply an exact correction for partial volume distortions. This approach allows the determination of an accurate arterial input function, which was previously validated against the reference standard of arterial blood sampling (16). Once the arterial input function was derived from the dynamic PET image sequence, parametric images representing MRGlc (in μmol/100 g/min) were calculated using the operational equation implemented for PET by Phelps et al. (18), based on the original equation developed by Sokoloff et al. (19) and the FDG tracer distribution between 40 and 60 min p.i. The quantification process is fully automated and was performed off-line on a dedicated PC workstation. The total processing time was ~6 h per subject.

### Quantitative Assessment of Abnormal Glucose Metabolic Rate in Epilepsy Patients

**Figure 1:**
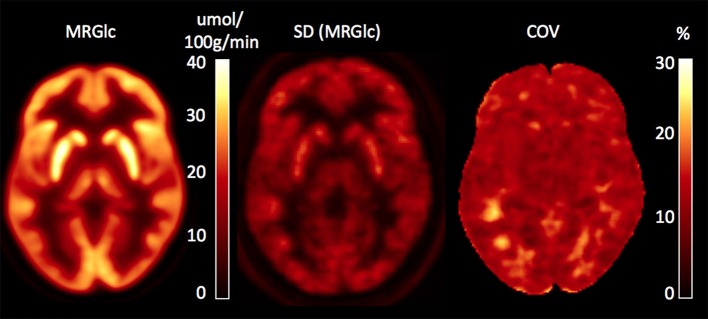
Database images in MNI space representing the mean, SD and coefficient of variation (COV) maps for absolute values of MRGlc. The COV map indicates a normal physiological variability of 10–20%.

In order to assess abnormal MRGlc areas in epilepsy patients, the recently described normative database of MRGlc images was used (16). The database includes 20 image volumes derived from a group of healthy controls (5M/5F, median age 27.1 years, IQR = 7.6 years) who were scanned twice using a test-retest design. As discussed in detail in our previous paper, we pooled data from both test and retest studies in order to create a normative database that accounts not only for CMRGlc variability due to between-subjects effects, but also accounts for changes in the subject's psychological state caused by uncontrolled fluctuations in brain activity. Spatially normalized MRGlc images were averaged to define a mean MRGlc (MRGlc_m_), a standard deviation (MRGlc_SD_) as well as the corresponding coefficient of variation (COV = SD/mean) image, constituting a normative database. Given the observed physiological variation (combined within and between subjects) in MRGlc values determined in the control group, the normal COV across the brain is in the range of 10–20% (Figure 1). The same approach was also used to spatially normalize MRGlc images obtained from ETLE patients. An abnormality map was then created for each patient by comparing the individual MRGlc image with the control mean (MRGlc_m_). Regions of significant hypo- or hyper-metabolism were defined as areas in the patient's abnormality map that exceeded ±2 SD (as defined in the MRGlc_SD_ image). Finally, these hypo/hyper-metabolic areas were projected back into the patient's native space and displayed onto the patient's own structural T1-w images. Quantitative readings (qPET) were then performed by viewing the patient's MRGlc images side-by-side with the hypo/hyper-metabolic areas overlaid onto the corresponding T1-w images. Quantitative analysis was performed by two collaborators (LS, OM) who were blinded to all clinical information about the patients.

### Statistical Assessment

In a clinical setting, the added value of a test is assessed based on whether patient management is affected. Our main objective was to determine whether fully quantitative analysis of FDG PET data based on advanced PET/MR scanner methodology is able to provide added value to the established clinical epilepsy work-up which might change the classification of patients with complex epilepsy pathology from being rejected as surgical candidates to being considered for further testing. Given the fact that our study focused on the added value associated with quantitative PET imaging in relation to the established clinical evaluation procedure of ETLE patient in the absence of surgical outcome data (due to the fact that all patients were rejected as surgical candidates based on seizure semiology, patient history, ictal and interictal scalp EEG and visual interictal PET/CT imaging), our results are necessarily descriptive. Thus, our primary outcome measure was the percentage of patients that was re-classified from being rejected to being considered for further testing. As a secondary outcome measure we also assessed the percentage of cases in which MRGlc maps may have generated additional information about possible underlying disease mechanisms or might have increased confidence in the original readings. Specifically, the added value provided by the abnormality maps is rooted in the sensitization of the reading physician to subtle abnormalities that might have not been appreciated during the initial clinical reading or in the confirmation of abnormalities that were initially detected but were considered marginal. To quantify the agreement between visual FDG PET/CT readings and MRGlc maps, we calculated the percentage of anatomical locations that were determined as abnormal by both methods against the total number of anatomical locations determined as abnormal by either method. Accordingly, an agreement score of 1.0 reflects perfect agreement (no additional new information, both methods determined the same abnormalities) whereas an agreement score of 0 indicates no overlap between the two methods (the two methods determine completely different sets of abnormalities). All values are expressed as mean ± SD.

## Results

Our dataset included complete clinical information and results of PET imaging for 15 patients with drug-resistant non-lesional ETLE. There were 7 females and 8 males with a median age of 26.5 years (IQR = 9.5 years). Clinical details about patients, including age at seizure onset, duration of disease, seizure frequency, results of Video-EEG monitoring, structural MRI and time to PET/MRI before last seizure are presented in Table 1. Although some of the patients showed temporal lobe abnormalities, they were chosen based on strong suspicion for the presence of extratemporal lobe epilepsy foci. All patients were originally rejected as surgical candidates based on consensus decision founded on semiology, patient history, visual FDG PET/CT imaging and both ictal and interictal scalp EEG.

**Table 1:** Clinical information for all patients.

**Patient no**.	**Age (years)**	**Gender**	**Age at seizure onset (years)**	**Duration of disease (years)**	**Seizure frequency**	**MRI**	**Clinical seizure semiology**	**Ictal EEG pattern**	**Interictal EEG**	**Time between last seizure and qPET (days)**
# 1	21	f	7	14	>1 per week	Negative	R hemispheric	R temporo-parietal	No IED	7
# 2	25	m	22	3	<1 per week	Negative	L frontal and temporo-parietal	bilaterally frontal	No IED	21
# 3	29	m	14	15	>1 per week	Negative	R temporal and L temporal	bilateral independent seizure pattern: R temporal and L hemispheric	L temporal	2
# 4	39	m	33	6	>1 per week	Negative	R hemispheric	R temporo-lateral/parietal	R temporal and R parietal	4
# 5	33	m	11	22	>1 per week	Negative	L hemispheric and R central	bilateral independent seizure pattern: L parietal and R central	No IED	>28
# 6	29	f	20	9	<1 per week	Negative	non-localizable	non-localizable	R and L temporal	>28
# 7	21	f	14	7	<1 per week	Negative	R hemispheric	R parieto-occipital	No IED	>28
# 8	22	m	8	14	>1 per week	Negative	R hemispheric	R temporal/parietal	R temporal	>28
# 9	47	m	3	44	>1 per week	Negative	frontal	Bilateral non-lateralized	R temporal	27
# 10	26	f	24	2	>1 per week	Negative	Non-localizable	L hemispheric	L parietal	2
# 11	46	m	35	11	<1 per week	Negative	Non-localizable	L frontal; L hemispheric	L hemispheric	>28
# 12	21	f	20	1	<1 per week	Negative	Non-localizable	L parietal	L parietal	>28
# 13	26	f	21	5	<1 per week	Negative	L hemispheric	L parietal and L frontal	L hemispheric	>28
# 14	29	f	11	18	>1 per week	Negative	R hemispheric	R frontal and bilateral frontal	R and L frontal	7
# 15	21	m	5	16	<1 per week	Negative	L hemispheric	Bilateral L lateralized	L hemispheric	3

*F, female; m, male; MRI, magnetic resonance imaging; IED, interictal epileptiform discharges; R, right; L, left*.

### Performance of qPET Analysis

With respect to the primary outcome measure, quantitative PET imaging did not provide sufficient added information to reverse the original clinical decision, which was to reject patients as surgical candidates. Overall, qPET demonstrated at least partial confirmation of prior clinical impression in 8/15 (53%) of cases, whereas in the remaining cases qPET and clinical readings either did not agree on the location of abnormality (4/15 = 27%) or both methods failed to detect any abnormalities (3/15 = 20%). Furthermore, in 5/15 (33%) cases, quantitative assessment yielded additional information about a potentially underlying disease mechanism (e.g., WM inflammation, thalamus involvement). Table 2 provides details with respect to the relationship between quantitative FDG PET analysis and pre-imaging visual FDG PET impression.

**Table 2:** Contribution of quantitative imaging to clinical decision.

**Patient no**.	**Clinical decision^**a**^**	**qPET localization**	**Overlap of qPET localization with clinical reading**	**Contribution of qPET to understanding disease**	**vPET localization**	**Added value of qPET to vPET localization**
# 1	R temporo-parietal	Hypo-metabolism R parietal inferior and R thalamus	Yes	Yes, involvement of thalamus	Hypo-metabolism R temporal and R inferior parietal	Yes
# 2	L fronto-temporo-parietal	**Hyper-metabolism R central and L precentral**	No	No	No abnormalities	Yes
# 3	Bilateral independent foci R temporal and L hemispheric	Hypo-metabolism L parietal and L temporal; **Hyper-metabolism bilateral in WM**	Partial	Yes, Inflammation of WM	Hypo-metabolism L temporal and L inferior parietal	yes
# 4	R temporal lateral/ parietal	Hypo-metabolism R temporal and L anterior insular	Partial	Yes, role of contralateral insula	Hypo-metabolism R temporal and R inferior parietal	Yes
# 5	Bilateral independent foci L parietal and R central	Hypo-metabolism bilateral in WM	No	No	No abnormalities	Yes
# 6	Non-localizable	No abnormalities	No	No	No abnormalities	No
# 7	R parieto-occipital	Hypo-metabolism R temporal and R superior parietal	Partial	Yes, area much more extended	Hypo-metabolism R temporal	Yes
# 8	R temporal-insular	Hypo-metabolism R temporal and insular	Partial	No	Hypo-metabolism R temporal and insular	No
# 9	frontal, non-localizable	Hypo-metabolism R mesiotemporal, R dorsolateral prefrontal, L thalamus	Yes	Yes, involvement of thalamus	Hypo-metabolism R temporal and frontolateral	Yes
# 10	L parietal	No abnormalities	No	No	No abnormalities	No
# 11	L frontal	Hypo-metabolism L frontal	Yes	No	Hypo-metabolism L frontal and R central	No
# 12	L parietal	No abnormalities	No	No	Hypo-metabolism L parietal and temporal	No
# 13	L temporo-parietal	No abnormalities	No	No	Hypo-metabolism L temporal anterior	No
# 14	R frontal	Hypo-metabolism R parietal	Partial	No	No abnormalities	No
# 15	L hemispheric	No abnormalities	No	No	No abnormalities	No

a *Based on results of scalp Video-EEG-monitoring (interictal and ictal EEG data and clinical semiology of seizures) and clinical FDG PET/CT*.

*R, right; L, left; WM, white matter; qPET, quantitative PET analysis; vPET, visual PET readings*.

*Detection of hyper-metabolic abnormalities are highlighted*.

**Figure 2:**
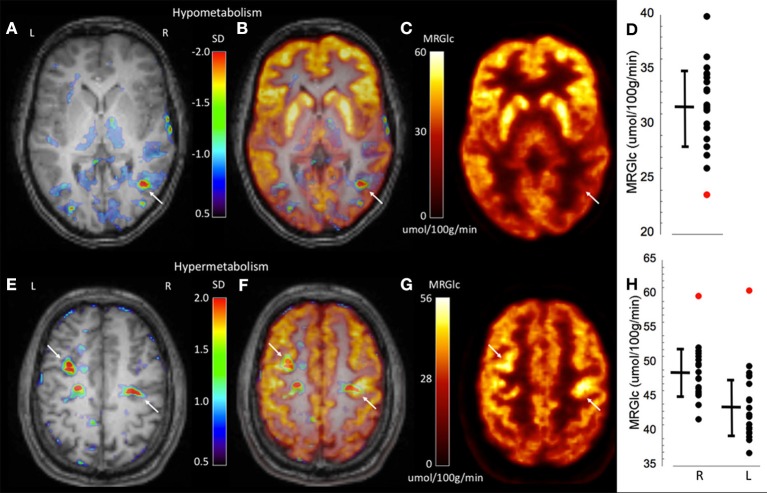
Representative images of two epilepsy patients (top row patient #1, bottom row patient #2) showing focally abnormal hypo- and hyper-metabolic areas. **(A)** Abnormality map overlaid onto the patient's structural MR scan shows a significant focal hypo-metabolic area (>2 SD of control mean) in the right parieto-temporal region (white arrow). The color scale represents the SD below normal mean MRGlc at the location of each individual voxel. **(B)** Overlay of the abnormality map onto the MRGlc and structural MR images. **(C)** Focally decreased MRGlc area in the right parieto-temporal lobe (white arrow). **(D)** Graph demonstrating decreased MRGlc in the significant abnormality area of the patient (red dot) in comparison to corresponding MRGlc values in the control group (black dots). **(E)** Abnormality map overlaid onto the patient's structural MR scan shows two significant focal hyper-metabolic areas (>2 SD of control mean) in the right central and left precentral region (white arrows). **(F)** Overlay of the abnormality map onto the MRGlc and structural MR images. **(G)** Focal hyper-metabolic areas in the right central and left precentral region. **(H)** Graph showing increased MRGlc values (red dots) in comparison to corresponding values derived from the normal control group.

To demonstrate the performance of quantitative analysis, Figure 2 shows representative images of two patients in whom focal hypo-metabolic (patient #1) and hyper-metabolic (patient #2) areas were detected using the proposed method. In patient #1, a focus in the right temporo-parietal lobe was suspected based on ictal clinical semiology, however corresponding MR images did not provide any indication for tissue abnormalities. Absolute quantification of MRGlc detected a hypo-metabolic area in the right parieto-temporal lobe that was >2 SD below normal mean MRGlc (Figure 2D). Interestingly, in this patient the abnormality map implied decreased metabolism in the ipsilateral thalamus (although not significant), thus, supporting a right hemispheric seizure onset (Figure 2A). In contrast to patients in whom quantitative analysis confirmed visual readings of hypo-metabolic areas, Figure 2E shows a patient (patient #2) with bilateral focal hyper-metabolic areas at the R central and L precentral locations that exceeded 2 SD above normal mean MRGlc (Figure 2H). These PET-defined abnormalities were not observed in the corresponding high-resolution MR images, suggesting a non-lesional cause of seizure onset. Moreover, these hyper-metabolic areas were not reported in the original PET reading, but were visually confirmed after detection by quantitative analysis. Of note, neither visual nor quantitative analysis detected any hypo-metabolic areas. This patient suffered from seizures that triggered loss of consciousness and semiology indicated bilateral involvement, which was supported by the quantitative analysis. Based on the clinical context, these abnormalities might indicate increased metabolic activity adjacent to the epileptic focus in order to inhibit activity in the seizure onset area. Nevertheless, the relevance of the observed hyper-metabolic areas during the ictal state in this patient remains unclear.

Several other cases deserve a closer inspection. In patient #4 visual analysis suggested a right-lateralized seizure onset in the temporo-parietal region, whereas qPET indicated bilateral seizure onset (R temporal and L anterior insula). In this particular patient the consensus decision during epilepsy conference was a unilateral right-sided seizure onset as indicated in the first column in Table 2 (“Clinical decision”). As the clinical decision is the basis for further management of this patient, the qPET finding of L anterior insula abnormality has to be regarded as a false positive finding. In patient #9, the clinical decision was a frontal, non-localizable seizure onset which was partially confirmed by qPET (R dorsolateral). In addition, qPET also suggested the involvement of the L thalamus which was not reported by visual analysis. Interestingly, this patient showed in addition to the extratemporal abnormalities also hypometabolic abnormalities in the R mesio-temporal lobe which were detected by both visual and qPET. Thus, this patient might suffer from both temporal as well as ETL epilepsy. Both ipsilateral as well as contralateral thalamic hypometabolism was reported in patients with temporal lobe abnormalities (20). Accordingly, it is possible that the contralateral thalamic abnormality in this patients was associated with the detected temporal lobe abnormality.

### Assessment of WM Abnormalities

**Figure 3:**
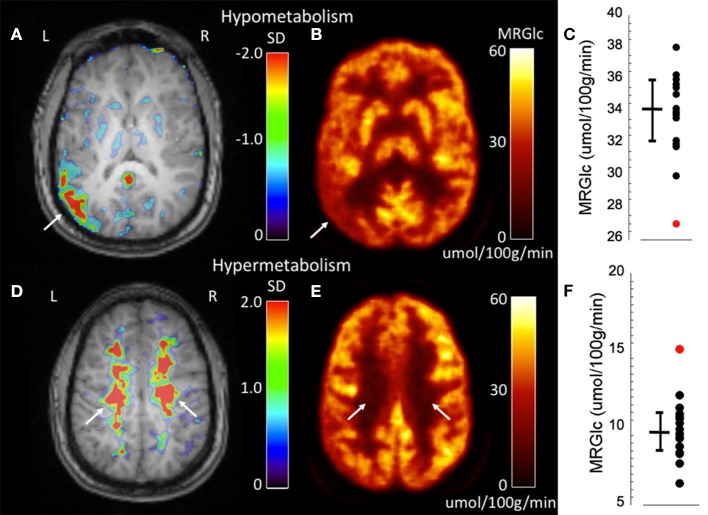
Representative images of an epilepsy patient (#3) showing both hypo- (top row) as well as hyper-metabolic (bottom-row) brain areas. **(A)** Abnormality map overlaid onto the patient's structural MR scan shows a significant focal hypo-metabolic area (>2 SD of control mean) in the left parieto-temporal region (white arrow). The color scale represents the SD below normal mean MRGlc at the location of each individual voxel. **(B)** Focally decreased MRGlc area in the left parieto-temporal lobe (white arrow). **(C)** Graph demonstrating decreased MRGlc in the significant abnormality area of the patient (red dot) in comparison to corresponding MRGlc values in the control group (black dots). **(D)** Abnormality map overlaid onto the patient's structural MR scan shows extensive significantly hyper-metabolic areas in the bilateral centrum semiovale (white arrows). **(E)** Large-scale increases of MRGlc in the bilateral centrum semiovale (white arrows). **(F)** Graph demonstrating significantly increased MRGlc in the centrum semiovale of the patient (red dot) in comparison to corresponding MRGlc values in the control group (black dots).

An advantage of absolute quantification is the assessment of extensive WM abnormalities that are difficult to appreciate during clinical readings. One of our patients (#3) showed seizures originating independently from the left and right hemisphere. Both the clinical PET readings as well as quantitative assessment identified a hypo-metabolic area in the left temporo-parietal cortex (Figures 3A–C). However, quantitative assessment of MRGlc also detected extensive hyper-metabolic areas in the WM (centrum semiovale) that suggest low-level inflammation of brain tissue (Figures 3D–F), as demonstrated previously in amyloid-positive patients with Alzheimer disease (21). This result indicates that both hypo- as well as hyper-metabolic areas can be present simultaneously in the epileptic brain, emphasizing the importance of absolute quantification in order to separate contributions stemming from different pathologies. Interestingly, this patient might be part of a familial (genetic) presentation of epilepsy as his mother, brother and cousin all suffer from epilepsy. The obtained quantitative data initiated further investigations in order to determine the cause for increased MRGlc in the WM.

### Agreement Between Quantitative and Visual PET Analysis

In order to determine to what extent results of quantitative analysis deviated from visual readings of FDG PET/CT images, the average agreement score between the two approaches was determined. The average agreement score was found to be 0.47 ± 0.35, which included 3 cases with perfect overlap (no abnormalities detected by both methods) and 3 cases with no overlap (abnormalities detected with quantitative analysis were read as normal visually).

Overall, in 12/15 (80%) of cases the two methods were correspondent and as a result qPET analysis either increased confidence in, or expanded upon, the original visual readings. In the remaining 3 cases, results obtained from the two methods differed as follows: in patient #2 qPET indicated bilateral focal hyper-metabolic areas at the R central and L precentral location which were not reported in the original PET readings, but were visually confirmed after detection by quantitative analysis and re-reading of semi-quantitative PET images. Moreover, in patient #5 qPET indicated bilateral hypo-metabolic areas in WM which were not reported by visual assessment and is not surprising, as abnormalities in the WM remain usually unreported during visual analysis. Finally, in patient # 12 visual assessment identified hypometabolic areas in the L parietal and L temporal lobe which were not confirmed by qPET analysis.

## Discussion

Our work demonstrates moderately improved characterization of glucose metabolic abnormalities in patients with non-lesional ETLE epilepsy based on absolute quantification of MRGlc in a clinical setting. We report here four main findings: quantitative assessment of MRGlc: (1) does not provide sufficient additional information that could justify re-classification of patients who have been rejected as surgical candidates based on established clinical work-up; (2) contributes to increased confidence in the original visual readings in ~50% of cases; (3) identifies hyper-metabolic areas that are rarely acknowledged in clinical readings but might reflect dynamic changes in brain networks that are directly associated with seizure generation and propagation; and (4) sets a limit to the sensitivity of absolute quantification to detect hypo- or hyper-metabolic areas in epilepsy patients that is caused by the relatively large physiological variation (10–20%) of MRGlc values in the normal population.

### Clinical Value of Quantitative FDG PET Analysis

Although our findings indicate that the inclusion of quantitative MRGlc maps into clinical management of ETLE patients does not have the capability of changing classification from being rejected to being considered as surgical candidates, it can provide added confidence with respect to the location of suspected seizure onset areas, especially in cortical regions that are subject to extensive gyral folding pattern. Visual readings of FDG PET images are heavily influenced by the assessment of tracer uptake asymmetries between homotopic cortical territories that are sensitive to physiological variation in hemispheric cortical folding. Despite the fact that overlay of high-resolution MR images onto FDG PET images might shed light on whether the asymmetrical FDG tracer activity is due to an unbalanced gyral folding pattern, high-resolution MR images are not always available and even if available, might not provide a conclusive answer. As a result, expert readers find themselves often in a situation to make a judgment call whether a particular asymmetry is “real” or whether it is merely an artifact due to physiological variability in cortical shape. Moreover, in the absence of an absolute scale, it is unclear whether the observed asymmetries represent abnormal decreases of tracer concentration in one hemisphere or alternatively abnormal increases in the contralateral hemisphere, especially in complex pathologies characterized by more extensive abnormalities where visual comparison against areas that are assumed to be not involved in the epileptogenic network (such as the brainstem or cerebellum) is difficult. Visual PET readings therefore tend to be conservative in order to keep the level of false positive findings at bay, accepting the unavoidable higher false negative rate. Accordingly, the role of quantitative PET analysis is to provide added information with respect to the identification of “suspicious” territories that warrant a closer inspection by the clinical reader. This approach results in greater confidence of those abnormalities that have been identified independently by both methods and sensitizes the clinical team to scrutinize areas that were not identified by both methods. In addition, this approach also encourages both identification and reporting of hyper-metabolic areas that might represent frequently spiking cortex prone to epileptiform activity.

Of special interest is the identification of abnormalities by quantitative analysis in two areas of the brain—the thalamus and white matter—that are difficult to evaluate by visual analysis alone. In our study, abnormalities in these areas were only detected by the quantitative analysis and the relevance of these findings remains unclear, although recent studies are beginning to address the contribution of these structures to ETLE pathologies (22). These studies have demonstrated significantly reduced thalamic gray matter densities as well as decreases in WM fractional anisotropy (FA, a measure of fiber tract integrity) in ETLE patients. It is well-accepted that seizure initiation and spread involves a complex interaction between the cortex and several nuclei of the thalamus (23). Specifically in ETLE, it is believed that various independent seizure foci in the cortex (each of which might give rise to different clinical manifestation) project to thalamic nuclei that organize the excitatory drive in an ictal discharge, from where then various pathways recruit new cortical regions into the seizure. In addition, frequent electrical discharges spreading along multiple pathways from the thalamic nuclei cause damage to major fiber tracts, leading to reduced axonal density and abnormalities in the myelin sheets (24). Although both thalamic as well as WM abnormalities have been frequently reported in idiopathic generalized epilepsies (25–27), they are rarely picked up by clinical FDG PET readings. Our results demonstrate that quantitative assessment of glucose metabolism is sensitive to thalamic and WM abnormalities, providing added information that increases the perception of subtle abnormalities during clinical readings.

Finally, our analysis included several cases where qPET and clinical readings did not agree on the location of abnormality. The two cases (#2 and #5 in Table 2) where qPET detected abnormalities which were initially not identified by visual readings included either hyper-metabolic cortical areas or bilateral hypo-metabolic areas in the WM. Visual detection of such abnormalities is problematic and regardless of whether these abnormalities are real or not, the observed mismatch initiated a review of these abnormalities and sensitized the reading physician to carefully re-evaluate the original readings. Our analysis also included two cases (#12 and #13 in Table 2) where visual analysis identified cortical abnormalities which were not detected by qPET. The reason for this mismatch might be a lower sensitivity of qPET for relatively subtle abnormalities as compared to visual assessment.

### Impact of PET/MR Imaging

FDG PET imaging is considered an important diagnostic tool that provides imaging clues with respect to epileptogenic brain areas. This is in addition to MRI that is used as the standard imaging method for the anatomical and functional assessment of the brain (28). Systemic combinations of PET and MR have been proposed first in the realms of pre-clinical imaging (29) and, as of 2006, are available also for imaging of patients (30, 31). However, in the absence of arterial blood sampling in clinical routine, PET-only based delineation of cortical abnormalities is entirely dependent on semi-quantitative measures, such as standardized uptake values (SUVs) or regional measurements of tracer concentration against normative values (32). Latest developments in PET/MR methodology have opened the possibility to derive an IDIF (16, 33, 34) that can be used in lieu of arterial blood samples to perform absolute quantification of MRGlc. We hypothesized that such clinical quantification might provide additional clues with respect to the location of primary and secondary epileptic foci, as it not only identifies hypo-metabolic areas, but also provides information about brain areas that are hyper-metabolic.

### Relevance of Hyper-Metabolic Areas in Non-lesional ETLE

To date, the relevance of hyper-metabolic areas that are present in the interictal state remains unclear and as a consequence, has been rarely reported. There are only a handful of studies that have addressed the significance of such hyper-metabolic areas in the context of epilepsy (13, 14). These investigators have speculated that such areas might represent either a pathophysiological state of neuronal hyperactivity that is predisposed to epileptic discharge generation, or a network of inhibitory circuits activated to prevent the spread of epileptic discharge originating from a localized epileptogenic zone (13, 15, 35). There is evidence for both of these scenarios: Bansal et al. (13) reported that ictal hypermetabolic areas correlate well with regions of high spike count (>10/min) as determined using intracranial EEG recordings and others have observed spike counts exceeding 44 per minute associated with interictal hyper-metabolism (36). Moreover, Juhasz et al. (35) demonstrated that more than half of the electrocorticography (ECoG defined) seizure onset areas are located in the cortex with normal- or hyper-metabolism, suggesting an extended network of hyper- and hypometabolic cortical areas in which reduced metabolism is associated with decreased synaptic activity and tonic hyperpolarization of neurons, protecting these cortical areas from ictal involvement. The functional isolation of epileptic foci from their surrounding neuronal connections may influence the excitability of the neuronal population and may prevent self-sustaining synchronized neuronal activity, as shown by successful suppression of focal epileptic activity by subpial transections both in animal models and in human epilepsy surgery of eloquent cortex (37, 38). Based on these observations, one can hypothesize that hypometabolic cortex without ictal electrophysiological involvement might represent regions with functional abnormality that are actually protected from participation in the seizure activity. Thus, the reliable detection of hyper-metabolic areas might allow non-invasive delineation of interictally spiking cortex that can be assessed in spatial relationship to cortical areas that represent a functional disconnection of the focus from surrounding synaptically connected areas.

### Limitations of Quantitative PET Analysis

The fully quantitative assessment of brain MRGlc provides valuable information about the regional metabolic state of brain tissue, but this advance in methodology brings its own set of issues that need to be carefully considered. Early studies that have investigated absolute cerebral MRGlc in control subjects have revealed a surprisingly large physiological variability that was in the range of 10–20%, even for large regions and the same subject being scanned only a few days apart (39, 40). Consequently, in the absence of improved data acquisition protocols that are able to standardize the brain state of the subjects under study, sensitivity to detect areas of significantly increased or decreased MRGlc will be relatively low, requiring about 25% deviations from baseline. This compares unfavorably with the visual assessment of regional asymmetries between homotopic brain areas, which can be quite easily detected at the 10–15% level. Thus, in order to improve the relevance of absolute quantification for the detection of epileptic foci, standardization of the subjects' brain state will be necessary. Unfortunately, it is currently unclear how such a standardization could be achieved.

Based on the above it is easy to conceptualize how changes in bodily energy level, visceral control as well as psychological factors such as mood and motivation might affect the intrinsic state expressed in the regional activity of the brain. Unfortunately, it is extremely difficult to standardize the psychological state of the brain across a group of control subjects, and it is practically impossible in a clinical environment (41). Conceptually, a coefficient of variation of about 5% has been shown to characterize physiological fluctuation in regional cerebral blood flow and is considered the lower limit of variability achievable (42). It is conceivable that future advances in our understanding of the underlying metabolic processes that cause the considerable fluctuation in MRGlc will help in designing PET protocols with physiological variability close to the lower limit of 5%.

### Other Limitations

A major limitation of our study is the paucity of true outcome data, usually assessed based on the presence/absence of seizures following surgical resection. Such data is obviously not available in patients who have not been found viable for surgery. Moreover, in patients who have been considered for surgery, placement of invasive electrodes, and selection of brain areas to be resected is never decided by one modality alone—it is reached through consensus derived from various sources, such as scalp EEG, semiology, patient history, PET, and MRI. As a result, even if PET imaging is suggestive of a distinct seizure focus, the patient might still not be recommended for surgical intervention. Accordingly, we propose that the relevance of qPET lies in increasing the confidence of visual readings, either by confirming or questioning the initial diagnosis. Moreover, in the absence of intracranial EEG electrode placement it is not possible to determine whether any of the observed abnormalities are true or not, however clinical decisions are made constantly based on such incomplete information. For this reason, we believe that it is reasonable to regard the consensus decision of the epilepsy conference as the de facto “ground truth.” A related issue is that of sample size. Our study group represents a clinically heterogenous group of non-lesional ETLE patients, each of whom exhibits a distinct clinical presentation that gives rise to a unique abnormality pattern. Considering the individual nature of each case, we believe that discussing each case on its own (with respect to the added value of qPET) is a meaningful approach. The same applies also for the control group, as one of the main outcomes of our paper is the realization of high physiological variability of MRGlc (both across time and across subjects) in the normal population, setting a limit to the sensitivity associated with absolute quantification.

## Conclusion

In a selected group of patients with non-lesional ETL epilepsy who were originally rejected as surgical candidates, quantitative PET imaging failed to demonstrate sufficient added information to reverse the original clinical decision. However, absolute MRGlc values did contribute to the detection of subtle hypo- or hyper-metabolic brain areas that are often missed in clinical readings in ETLE patients, thereby increasing confidence in visual PET readings. Moreover, the high normal physiological variability of glucose metabolic rates limits the sensitivity of quantitative analysis. Taken together, our investigation documents both the potential as well as the limitations of absolute quantification based on advanced simultaneous PET/MR imaging for the management of non-lesional ETL epilepsy cases in clinical routine.

## Data Availability Statement

The datasets generated for this study are available on request to the corresponding author.

## Ethics Statement

The study was approved by the Ethics Committee of the Medical University of Vienna and was performed in accordance with the Declaration of Helsinki (1964) including current revisions, the Austrian Drug Law and the GCP guidelines of the European Commission. Written informed consent was obtained from all subjects prior to the examinations.

## Author Contributions

TT-W and EP: design and conceptualized study, analyzed the data, study supervision, and drafted the manuscript for intellectual content. OM: design and conceptualized study, analyzed the data, and drafted the manuscript for intellectual content. LS: analyzed the data and drafted the manuscript for intellectual content. SA-W: analyzed the data with respect to clinical assessment of patients. TB: design and conceptualized study, study supervision, and drafted the manuscript for intellectual content. MH and RL: drafted the manuscript for intellectual content. AH: provided expertise with MR navigator sequences. GK: provided expertise with assessment of MRI in epilepsy patients. E-MK: provided expertise with respect to clinical assessment of epilepsy patients. MM: design and conceptualized study. MP: provided expertise with regard to ethical issues and analyzed the data. IR: design and conceptualized study with respect to methodological aspects of PET/MR imaging. LR: provided expertise with respect to quantitative analysis of multimodality data. WW: design and conceptualized study and drafted the manuscript for intellectual content.

### Conflict of Interest

The authors declare that the research was conducted in the absence of any commercial or financial relationships that could be construed as a potential conflict of interest.
